# Safety and effectiveness of all-oral and injectable-containing, bedaquiline-based long treatment regimen for pre-XDR tuberculosis in Vietnam

**DOI:** 10.3389/fphar.2022.1023704

**Published:** 2022-10-14

**Authors:** Thi Mai Phuong Nguyen, Binh Hoa Nguyen, Thi Thanh Thuy Hoang, Hoang Anh Nguyen, Dinh Hoa Vu, Mai Hoa Nguyen, Bao Ngoc Nguyen, Tom Decroo, Viet Nhung Nguyen

**Affiliations:** ^1^ Vietnam National Lung Hospital, Hanoi, Vietnam; ^2^ National Drug Information and Adverse Drug Reaction Monitoring Centre, Hanoi University of Pharmacy, Hanoi, Vietnam; ^3^ School of Pharmacy, Memorial University of Newfoundland, John’s, NL, Canada; ^4^ Institute of Tropical Medicine Antwerp, Antwerp, Belgium

**Keywords:** pre-XDR TB treatment, all oral regimen, injectable secondline drug, QT prolongation, bedaquline

## Abstract

**Background:** The World health organization (WHO) recently recommended standardized all-oral shorter regimens for rifampicin resistant Tuberculosis (RR-TB). For highly resistant Tuberculosis patients such as pre-XDR-TB: RR-TB plus additional resistance to fluoroquinolones (FQ), the 6–9-months bedaquiline (bedaquiline)-based regimens or BDQ-based long regimens are recommended. The role of second-line injectable (SLI) drugs in the treatment of drug resistant TB is restricted because of safety concerns. Nevertheless, it is not well-known how all-oral long regimens (BDQ-long) perform compared to SLI-containing long regimens (BDQ/SLI-long) in terms of safety and effectiveness among patients with highly resistant TB.

**Method:** A prospective observational cohort of patients with RR-TB additionally resistant to fluoroquinolones and/or second-line injectable, treated with either BDQ-long or BDQ/SLI-long regimens according to the guidance of the National Tuberculosis Program of Vietnam, enrolled between December 2015 and June 2017.

**Results:** Of 99 patients enrolled, 42 (42%) patients were treated with BDQ-long and 57 (57%) with BDQ/SLI-long. More than 85% of patients were previously exposed to both FQ and SLI. FQ and SLI resistance were confirmed in 28 (67%) and 41 (98%) in the BDQ-long cohort and 48 (84%) and 17 (30%) in the BDQ/SLI-long cohort, respectively. Treatment success was achieved among 29 (69%) and 46 (81%) patients on the BDQ-long and BDQ/SLI-long regimen, respectively (*p* = 0.2). For both regimens, median time to first smear/culture sputum conversion was 2 months. All patients experienced at least one adverse event (AE) and 85% of them had at least one severe Adverse events. The median time to a first severe adverse event was 2 months. Among patients treated with BDQ-long a higher proportion of patients had three QT-prolonging drugs in the regimen (26.2% versus 7.0%; *p* = 0.009). The severe prolonged QTcF was observed in 22 (52.4%) and 22 (38.6%) patients on BDQ-long and BDQ/SLI-long, respectively. Overall, 30 (30%) patients had to either temporary or permanently discontinued or more TB drugs due to AEs.

**Conclusion:** Treatment success was similar for both all-oral and SLI-containing BDQ-based long regimens in highly resistant TB patients. Both regimens had a similar high frequency of AEs. For both BDQ-long and BDQ/SLI-long regimens active AEs monitoring is essential.

## Introduction

Tuberculosis (TB) remains one of the most frequent, transmissible, life-threatening diseases globally, with high mortality and morbidity. Furthermore, the emergence of rifampicin resistant TB (RR-TB) has threatened global efforts in ending the disease. Treatment of RR-TB, especially in patients with more advanced resistance patterns such as pre-extensively drug-resistant tuberculosis (pre-XDR-TB) had poor outcomes until the recent introduction of bedaquiline (Abubakar et al., 2021). According to 2020 World Health Organization (WHO)’s global TB report, about 57% of patients with RR-TB were successfully treated and, compared to 47% of patients with pre-XDR-TB (World Health Organization, 2020). Vietnam is one of 30 high TB burden countries in the world, with high rates of RR-TB. In 2019, there were an estimated 170,000 new TB and 8,400 new RR-TB cases (World Health Organization, 2020). The prevalence of FQ resistance among patients with RR-TB was 16,7% (Nhung et al., 2015).

Over the last decade, WHO has issued a number of new guidelines for the management of RR-TB in order to improve the outcome and tolerability of RR-TB treatment regimens (World Health Organization, 2013; World Health Organization, 2014; World Health Organization, 2015; World Health Organization, 2016a; World Health Organization, 2016b; World Health Organization, 2020; Mirzayev et al., 2021; World Health Organization, 2022). RR-TB treatment regimens recommended by WHO have evolved from long (more than 18 months) injectable-containing regimens to shorter all-oral regimens (using bedaquiline [BDQ] instead of the injectable drugs). The most recent guidelines recommend the use of either 9–11-months all-oral BDQ-based regimen or a novel 6–9-months treatment regimen, which includes BDQ and a new drug, pretomanid (World Health Organization, 2022). For pre-XDR-TB patients, the novel 6–9 -month BPaL (BDQ, pretomanid and linezolid) regimen showed to be effective in a one-arm trial (Conradie et al., 2020) and has been recommended by WHO in the updated guidelines (World Health Organization, 2022). However, patients who were previously exposed to any component of BPaL are not eligible for this regimen. Additionally, given that pretomanid is not yet available in most settings, BDQ-based long regimens are recommended for these highly resistant patients (World Health Organization, 2020).

Since BDQ was introduced as a new and highly potential anti-TB drug (World Health Organization, 2013) a number of studies have been conducted to assess the outcomes of BDQ-based regimens for the treatment of drug-resistant TB. The majority of these studies were about RR-TB treatment while some focused-on smaller cohorts of pre-XDR-TB patients (Hatami et al., 2022). Overall, BDQ-containing regimens showed high culture conversion and treatment success rate among drug-resistant TB patients (Hatami et al., 2022). In its 2020 consolidated guidelines, WHO recommended the use of BDQ as a core drug in all-oral regimens to optimize treatment outcomes and minimize the toxicity of injectable agents (World Health Organization, 2020). However, in patients with more advanced resistance patterns (pre-XDR-TB), the choice of effective drugs is limited. Second-line injectables (SLIs) might still be an option as companion drugs to support and protect BDQ in the regimen (van Deun et al., 2018). Moreover, in drug-resistant TB patients with limited treatment options, the exclusion of injectable drugs may lead to a combination of drugs with a similar toxicity profile, for example when multiple QT prolonging drugs, such as fluoroquinolones, clofazimine, and bedaquiline, are used in the same regimen (Brust et al., 2021; Hughes et al., 2022). A number of previous studies described the effectiveness of BDQ-based regimens, with or without SLIs, but only treatment outcomes were assessed (Hatami et al., 2022). How all-oral long regimens (BDQ-long) perform compared to SLI-containing long regimens (BDQ/SLI-long) in terms of safety was less well described. To our best knowledge, no comparison has been made between all-oral BDQ-based and injectable-containing BDQ-based regimens in terms of both safety and effectiveness.

In Vietnam, in addition to standardized regimens for RR-TB patients, individualized long regimens were indicated for patients with pre-XDR-TB and patients who did not tolerate one ore more components of the standardized RR-TB regimen. This study aimed to describe the effectiveness and safety of individualized BDQ based long regimens with and without SLI drug on pre-XDR-TB patients.

## Methods

### Study design

This prospective observational cohort study invovled pre-XDR-TB patients treated with BDQ-based long regimen in three sites including Ha Noi, Ho Chi Minh city and Can Tho between December 2015 and June 2017 under the treatment guidance of National Tuberculosis Program of Vietnam (NTP).

### Setting and study population

Vietnam is a high TB and RR-TB burden country. In 2016, there were an estimated 126,000 new TB and 5,500 new RR-TB cases nationally, of which, only 2,450 RR-TB cases were enrolled on treatment. This study included RR-TB patients over 18 years of age, who had TB additionally resistant to either FQ (pre-XDR-TB) or SLI or both. Exclusion criteria included being at risk of cardiovascular complications (QTcF >500 ms), having end-stage of liver or renal diseases, being pregnant, or being a nursing mother.

### RR-TB and pre-XDR-TB management

Xpert MTB/RIF (Cepheid Inc, Sunnyvale, CA, United States ) was used for the diagnosis of RR-TB. Once diagnosed, RR-TB patients were screened for resistance to FQ by either genotypic (GenoType Hain MTBDRsl (Nehren, Germany); second-line line probe assay) or phenotypic drug susceptibility testing (DST). In addition, other drugs resistance including firstline drugs such as isoniazid, ethambutol, streptomycin, and SLIs including amikacin, capreomycin and kanamycin, were also assessed at baseline. After assessing eligibility, patients were registered and started on either the BDQ-long or BDQ/SLI-long treatment regimen, following WHO and national Programmatic Management of Drug resistant TB guidelines. In Vietnam, when this study was conducted, there were no short, standardized treatment options for pre-XDR-TB and/or SLI-resistant RR-TB. Such patients were put on individualized long regimens without new drugs (BDQ, delamanid or pretomanid). Only at our three study sites, BDQ-based long regimens were available. Based on the patients’ treatment history and DST profile, the National Clinical Committee decided whether to put patients on the BDQ-long or BDQ/SLI-long regimen. In most cases, the patients were put on the BDQ-long regimen if their DST results showed resistance to secondline injectables. Nevertheless, 17/57 patients with initial resistance to one SLI but susceptible to another SLIs on phenotypic DST, were treated with the SLI (kanamycin or capreomycin) for which susceptibility had been shown (BDQ/SLI-long). For both groups, the total treatment duration was 20 months. SLI were provided during 8 months in injectable-containing regimen group. BDQ was used in 24 weeks. In case bacteriological and clinical response to treatment was poor, the use of BDQ was prolonged with an additional 4–8 weeks, following the decision of the National Clinical Committee. Patients were followed up monthly during treatment (clinical examination, smear, liquid culture BACTEC MGIT 960, chest Xray and other tests). ECG monitoring was three times per week during the first 2 weeks, once per week in the following 2 weeks and then monthly until the end of treatment (see Supplementary Table S1 for a detailed monitoring schedule). Patients were hospitalized during the first 1–2 months, then discharged for ambulatory management at district or commune levels. Adverse events (AEs) were managed and reported monthly following the cohort event monitoring (CEM) protocol as part of a prospective, observational, cohort study of AEs (Bao Ngoc et al., 2021; World Health Organization, 2012).

### Study variables and definition

Exposure variables included patient age, sex, body mass index, resistance profile, previous TB drugs exposure, HIV and other co-morbidity status, and treatment regimen. Monthly sputum smear and culture results were used to determine the month of conversion. Based on WHO and national guidelines, treatment outcomes were grouped as favorable (cured or treatment completed) and unfavorable (treatment failure, died, or lost-to-follow-up [LTFU]). For the safety analysis, all abnormal clinical symptoms and test results were recorded and reported during treatment using CEM reports. Grading of AEs was based on the “Table of Grading the Severity of Adult and Pediatric Adverse Events, version 2.0” (November 2014) of the U.S National Institute of Allergy and Infectious disease (US Department of Health and Human Services et al., 2014). Grade 3 and 4 adverse events were reported as severe AEs. Serious AEs (SAEs) included any death, hospitalization, life-threatening AE, permanently disability or any grade 4 AEs. Severe prolonged QTcF was defined as QTcF >500 ms or an increase of 60 ms compared to the baseline QTcF value. To assess potential drug-drug interactions that can cause QT prolongation we counted the number of drugs included in each of the regimens that were also listed on the CredibleMeds’ QT drugs list (Woosley et al., 2022).

### Data collection and analyses

Pre-designed paper forms were developed to collect data from the patient’s medical records, including baseline patient information form; monthly clinical and bacteriological follow-up forms; and CEM forms. Then, the data collected was entered in the Microsoft Access 2013 database for further analysis. Frequencies and proportions were used to summarise categorical variables; medians and interquartile ranges were used to summarise continuous variables. The chi-square test was used to assess associations between exposure and outcome variables, a multivariable regression model was used to find predictors of having an unfavorable outcome. We used Kaplan Meier curves to visualize the probability of events (time to the first severe adverse event, time to drug discontinuation and time to sputum smear/culture conversion) during treatment. The Log-rank test was conducted to compare the time-to-event between different groups. A *p*-value less than 0.05 was considered statistically significant. Data analyses were performed using software R 3.4.4.

### Ethics approval

Ethical approval of this study was obtained from the Independent Ethic Committee of the Ministry of Health and National Lung hospital in Vietnam. Written informed consent was obtained from all studied patients.

## Results

### Patient characteristics

Baseline characteristics of 99 eligible patients are shown in Table 1. Of those, 42 (42.4%) were on the BDQ-long and 57 (57.6%) were on the BDQ/SLI-long regimens, respectively. The mean age of patients was 43.5 years. More than two thirds were male. In both groups, the initial treatment regimen contained a median of 5 (interquartile range [IQR] = 5–6) anti TB drugs. Overall, 76 (76.8%) patients had confirmed resistance to FQ (pre-XDR-TB), with 66.7% in the BDQ-long and 84.2% in the BDQ/SLI-long regimen group, respectively. The majority of patients were exposed previously to FQ (86.9%) and SLI (86.7%). A baseline positive culture was recorded in 71.4% of patients (*n* = 30) treated with BDQ-long and 80.7% of patients (*n* = 47) treated with the BDQ/SLI-long regimen.

**TABLE 1:** Patient characteristics by regimen.

Patient characteristics	Total (*N* = 99)		BDQ-long (*n* = 42)		BDQ/SLI-long (*n* = 57)		*p* value
	Mean	SD	Mean	SD	Mean	SD	
Age (year)	43.5	(13.9)	42.8	(10.7)	44.0	(15.9)	0.673
Body mass index (kg/m^2^)	18.4	(3.3)	18.3	(3.0)	18.4	(3.6)	0.828
Follow-up time (months)	17.4	(4.9)	16.7	(5.3)	17.8	(4.7)	0.292
	***n***	**(%)**	***n***	**(%)**	***n***	**(%)**	
Male	71	(71,7)	29	(69.0)	42	(73.7)	0.613
Resistance to any SLID	58	(58.6)	41	(97.6)	17	(29.8)*	<0.001
Resistance to FQ	76	(76.8)	28	(66.7)	48	(84.2)	0.041
Previous SLID treatment	86	(86.7)	37	(88.1)	49	(86.0)	0.756
Previous FQ treatment	85	(86.9)	36	(85.7)	49	(86.6)	0.972
Asthenia	23	(23,2)	12	(28.6)	11	(19.3)	0.280
Smoking	12	(12.1)	5	(11.9)	7	(12.3)	0.955
Alcohol dependence	5	(5.1)	2	(4.8)	3	(5.3)	1.000
Co-morbidities							
At least one co-morbidity	43	(43.4)	17	40.5	26	45.6	0.610
Diabetes	22	(22.2)	10	(23.8)	12	(21.1)	0.744
Peptic ulcer	9	(9.1)	2	(4.8)	7	(13.5)	0.294
Hepatitis	7	(7.1)	4	(9.5)	3	(5.3)	0.453
Osteoarthritis	5	(5.1)	1	(2.4)	4	(7.0)	0.392
Anemia	5	(5.1)	2	(4.8)	3	(5.3)	1.000
COPD	5	(5.1)	1	(2.4)	4	(7.0)	0.392
HIV infection	3	(3.0)	2	(4.8)	1	(1.8)	0.573
Renal failure	2	(2.0)	1	(2.4)	1	(1.8)	1.000
Initial Treatment Regimens							
Secondline injectables	57	(57.6)	0	(0.0)	57	(100.0)	NA
Kanamycin	34	(34.3)	0	(0.0)	34	(59.6)	NA
Capreomycin	23	(23.2)	0	(0.0)	23	(40.4)	NA
Bedaquiline	99	(100.0)	42	(100.0)	57	(100.0)	NA
Levofloxacin	16	(16.2)	10	(23.8)	6	(10.5)	0.076
Linezolid	58	(58.6)	36	(85.7)	22	(38.6)	<0.001
Clofazimine	86	(86.9)	41	(97.6)	45	(78.9)	0.007
Cycloserin	24	(24.2)	8	(19.0)	16	(28.1)	0.301
Pyrazinamide	90	(90.9)	40	(95.2)	50	(87.7)	0.294
Prothionamide	51	(51.5)	21	(50.0)	30	(52.6)	0.796
P-aminosalicyclic acid	34	(34.3)	20	(47.6)	14	(24.6)	0.017
Ethambutol	14	(14.1)	6	(14.3)	8	(14.0)	0.972
High dose Isoniazid	5	(5,1)	0	(0.0)	5	(8.8)	0.070
Potential QT prolongation drug—drug interactions							
LFX_CFZ_BDQ	15	(15.2)	11	(26.2)	4	(7.0)	0.009
LFX_BDQ	19	(19.2)	12	(28.6)	7	(12.3)	0.042
CFZ_BDQ	88	(88.9)	41	(97.6)	47	(82.5)	0.022
Microbiology							
Positive AFB	60	(60.6)	25	(59.5)	35	(61.4)	0.850
Positive culture	76	(76.8)	30	(71.4)	46	(80.7)	0.280

SD, standard deviation; SLI, second-line injectable; FQ, fluoroquinolone; COPD, chronic obstructive pulmonary disease; HIV, human immunodeficiency virus; WHO, world health organization; BDQ, bedaquiline; LFX, levofloxacin; CFZ, clofazimine; AFB, Acid-fast bacillus.

* 17/57 patients with initial resistance to one SLI, but susceptible to another SLI, were treated with the SLI, for which susceptibility had been shown.

Eleven (26.2%) patients in all-oral regimen group had the combination of BDQ with both levofloxacin (LFX) and clofazimine (CFZ) in the regimen while only 4 (7.0%) of patients in the other group had BDQ plus both drugs (*p* = 0.009).

### Treatment outcome

**FIGURE 1:**
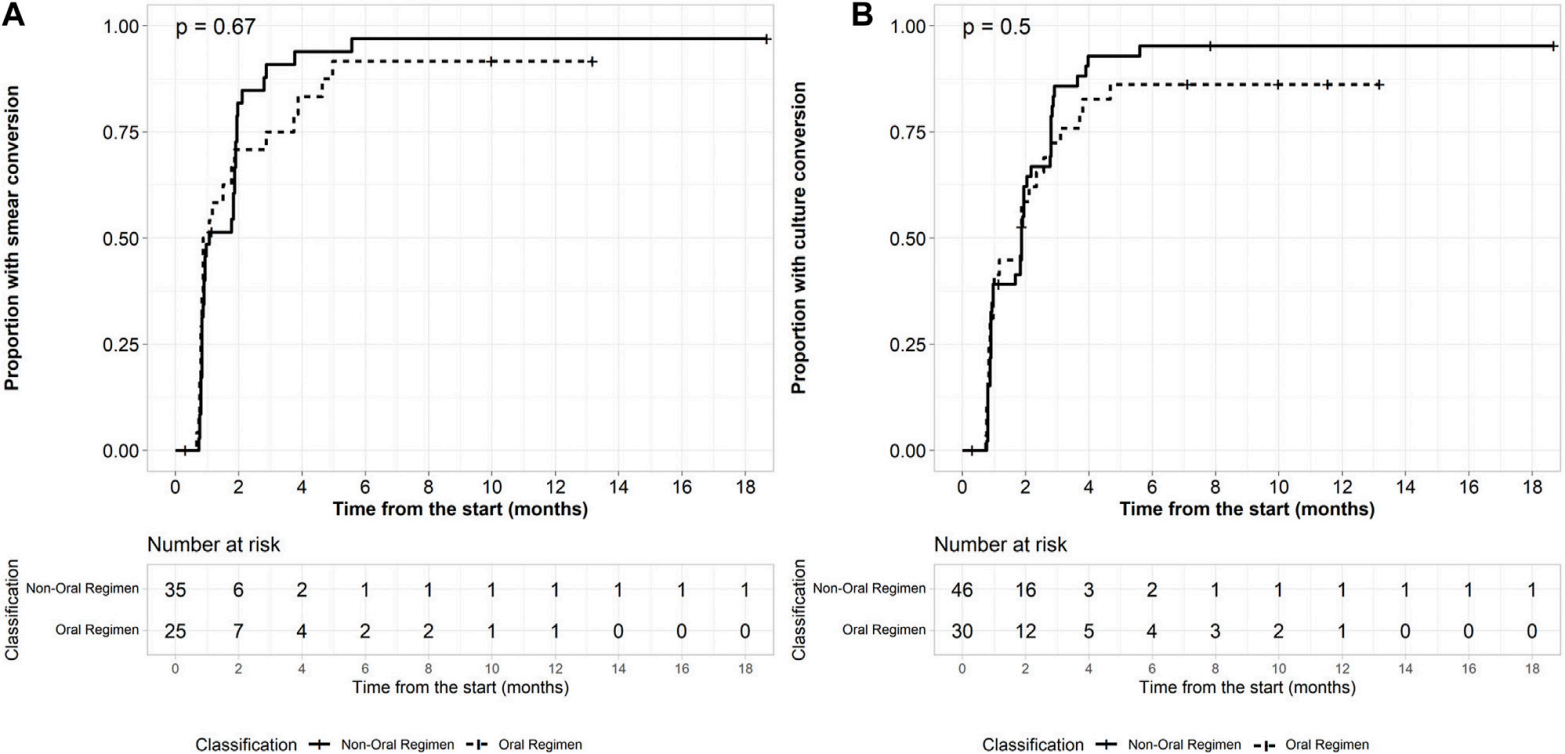
Baseline load predicts conversion. **(A)** Time to smear conversion in patients with positive microbiology **(B)** Time to culture conversion in patients with positive microbiology.

Bacteriological response was assessed in 76 patients with a positive culture sputum at baseline. Culture conversion at 2 months was achieved in 18 of 30 (60.0%) patients on BDQ-long and 30 of 46 (65.2%) patients on BDQ/SLI-long (*p* = 0.5). After 6 months of treatment, 86.7% (*n* = 26) and 95.7% (*n* = 44) patients culture converted in the BDQ-long and BDQ/SLI-long cohort (Figure 1). Treatment success was achieved among 29 (69.0%) and 46 (80.7%) patients of the BDQ-long and BDQ/SLI-long cohort (Table 2). The proportion with a favorable outcome was not significantly different between the 2 treatment groups (*p* = 0.2). No predictors having significant association with the favorable/unfavorable outcomes were found.

**TABLE 2:** Treatment outcome of BDQ-based regimens in the treatment of pre-XDR-TB.

	BDQ-long (*N* = 42)		BDQ/SLI—long (*N* = 57)		Total (*N* = 99)	
	*n*	(%)	*n*	(%)	*n*	(%)
Favorable outcome	29	(69.0)	46	(80.7)	75	(75.8)
Cured	24	(57.1)	38	(66.7)	62	(62.6)
Completed	5	(11.9)	8	(14.0)	13	(13.1)
Unfavorable outcome	13	(31.0)	11	(19.3)	24	(24.2)
Failure	1	(2.4)	1	(1.8)	2	(2.0)
Lost to follow up	8	(19.0)	7	(12.3)	15	(15.2)
Death	4	(9.5)	3	(5.3)	7	(7.1)

### Adverse events

**FIGURE 2:**
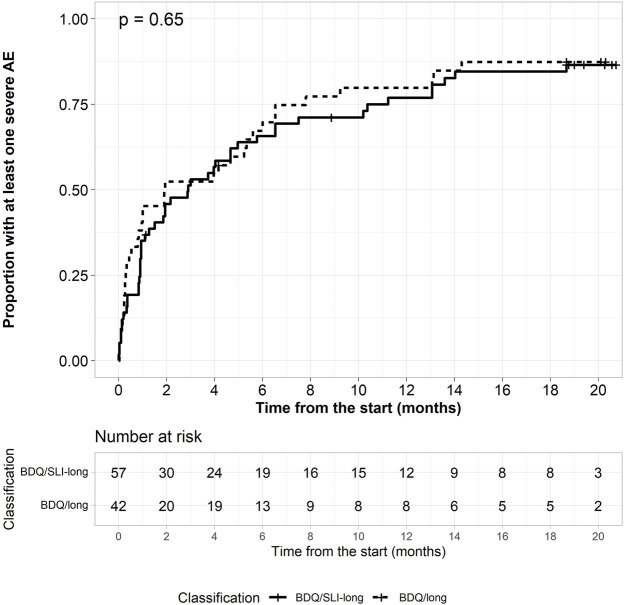
Kaplan-Meier plot for time to the first severe adverse event.

All 99 (100%) patients experienced at least one AE (grade 1–4) during their treatment, with a median of 7 (IQR: 5–10) AEs per patients. The proportion of patients having at least one severe AE (grade 3–4) was 85.7% (36/42) in the BDQ-long and 84.2% (48/57) in the BDQ/SLI-long cohort (Table 3). QT prolongation, metabolic disorders (hyper/hypoglycemia, hyperuricemia), liver function disorder, and electrolyte abnormalities were the most common severe AEs. There was no significant difference between the 2 regimens in relation to these abnormalities. Severe prolonged QTcF (defined as QTcF >500 ms or an increase of 60 ms compared to baseline) appeared in 44 (44.4%) patients. It occured in 22 (52.4%) patients treated with BDQ-long and 22 (38.6%) patients treated with the BDQ/SLI-long regimen. About half of patients with a first severe AE experienced it during the first 2 months of treatment (Figure 2). Serious AEs (SAE) were observed in 16 (38%) and 28 (49%) of those on BDQ-long and BDQ/SLI-long, respectively (*p* = 0.28) (Supplementary Table S2).

**TABLE 3:** Number of patients with severe AE by regimen.

	Total (*N* = 99)		BDQ-long (*N* = 42)		BDQ/SLI-long (*N* = 57)		*p* value
	*n*	(%)	*n*	(%)	*n*	(%)	
At least one severe AE	84	(84.8)	36	(85.7)	48	(84.2)	0.841
At least two severe AE	51	(51.5)	18	(42.9)	33	(57.9)	0.139
At least three severe AE	22	(22.2)	10	(23.8)	12	(21.1)	0.740
Heart rate disorders (prolonged QTcF)	44	(44.4)	22	(52.4)	22	(38.6)	0.172
Metabolic disorders	32	(32.3)	12	(28.6)	20	(35.1)	0.493
Hyperglycemia	18	(18.2)	8	(19)	10	(17.5)	0.842
Hypoglycemia	4	(4)	2	(4.8)	2	(3.5)	1.000
Hyperuricemia	15	(15.2)	5	(11.9)	10	(17.5)	0.238
Liver and biliary disorders	27	(27.3)	13	(31.0)	14	(24.6)	0.480
Increased direct bilirubin	23	(23.2)	10	(23.8)	13	(22.8)	0.920
Hepatitis	11	(11.1)	6	(14.3)	5	(8.8)	0.520
Electrolyte disturbances	18	(18.2)	8	(19.0)	10	(17.5)	0.841
Hypocalcemia	6	(6.1)	2	(4.8)	4	(7)	0.700
Hypomagnesemia	4	(4)	2	(4.8)	2	(3.5)	1.000
Hyperkalemia	2	(2)	1	(2.4)	1	(1.8)	1.000
Hypokalemia	8	(8.1)	4	(9.5)	4	(7)	1.000
Hematologic disorders	10	(10.1)	6	(14.3)	4	(7)	0.315
Gastrointestinal disorders	6	(6.1)	3	(7.1)	3	(5.3)	1.000
Vision disorders	6	(6.1)	3	(7.1)	3	(5.3)	1.000
Renal disorders (increased creatinine)	4	(4)	0	(0.0)	4	(7)	0.135
Hearing disorders	3	(3)	0	(0.0)	3	(5.3)	0.260
Psychiatric disorders	2	(2)	0	(0.0)	2	(3.5)	0.506
Any neurological disorders	2	(2.0)	0	(0.0)	2	(3.5)	0.506
Central nervous system disorders	1	(1)	0	(0.0)	1	(1.8)	1.000
Peripheral neuropathy	1	(1)	0	(0.0)	1	(1.8)	1.000
Muscoloskeletal disorders (arthralgia)	1	(1)	0	(0.0)	1	(1.8)	1.000
Anaphylactic reactions	1	(1)	0	(0.0)	1	(1.8)	1.000

AE, adverse event; AST, aspartate aminotransferase; ALT, alanine aminotransferase; QTcF, QT, interval corrected for heart rate using Fridericia’s formula.

Overall, 30 (30.3%) patients had to either temporary or permanently discontinue one or more TB drugs due to AE. Of group A drugs, linezolid (LZD) was discontinued most often: in 14.5% (11/76) patients having this drug in their regimen, compared to 7.1% (7/99) for bedaquiline and 3% (3/99) for fluroquinolone. Other drugs which were temporarily or permanently stopped in more than 13% of patients included ethambutol (14.3%, 2/14), SLI (14%, 8/57), cycloserine (13.5%, 5/37), p-aminosalysilic acid (13.3%, 6/45), and prothionamide (13.2%, 7/53) (Table 4). Regarding dose adjustment, 7 patients needed to reduce the dose of SLI because of electrolyte disturbances, increased creatinine and amylase, while the dose of LZD was reduced in 2 patients with hematologic disorders.

**TABLE 4:** Consequences of adverse events.

	Temporary or permanent		Permanent		Reasons for permanent discontinuation
	*n*	(%)	*n*	(%)		
Group A					
BDQ (*n* = 99)	7	(7.1)	3	(3.0)	Prolonged QTcF (*n* = 1), Gastrointestinal disorders (*n* = 1), Hepatitis (*n* = 1)
FQ (*n* = 21)	1	(4.8)	0	(0.0)	No
LZD (*n* = 76)	11	(14.5)	4	(5.3)	Prolonged QTcF (*n* = 1), Visual impairment (*n* = 2), Hepatitis (*n* = 1)
Group B					
CFZ (*n* = 94)	10	(10.6)	2	(2.1)	Prolonged QTcF (*n* = 1), Visual impairment (*n* = 2)
CS (*n* = 37)	5	(13.5)	2	(5.4)	Central nervous system disorders (*n* = 1), Psychiatric disorders (*n* = 1), Anaphylactic reactions (*n* = 1)
Group C					
PZA (n = 92)	6	(6.5)	4	(4.3)	Increased AST (*n* = 1), Gastrointestinal disorders (*n* = 1), Arthralgia (*n* = 1), Hepatitis (*n* = 2), Increased ALT (*n* = 1)
PTO (*n* = 53)	7	(13.2)	4	(7.5)	Increased AST (*n* = 1), Gastrointestinal disorders (*n* = 2), Psychiatric disorders (*n* = 1), Hepatitis (*n* = 1), Increased ALT (*n* = 1)
PAS (*n* = 45)	6	(13.3)	3	(6.7)	Gastrointestinal disorders (*n* = 2), Arthralgia (*n* = 1), Hepatitis (*n* = 1)
EMB (*n* = 14)	2	(14.3)	1	(7.1)	Gastrointestinal disorders (*n* = 1)
hINH (*n* = 12)	0	(0.0)	0	(0.0)	No
SLI (*n* = 57)	8	(14.0)	3	(5.3)	Hypokalemia (*n* = 1), Increased creatinine (*n* = 2), Peripheral neuropathy (*n* = 1), Hypomagnesemia (*n* = 1)

BDQ, bedaquiline; FQ, fluoroquinolone; LZD, linezolid; CFZ, clofazimine; CS, cycloserin; PZA, pyrazinamide; PTO, prothionamide; PAS, acid para-aminosalicylic; EMB, ethambutol; hINH, high dose isoniazid; SLI, second line injectable; AST, aspartate aminotransferase; ALT, alanine aminotransferase; QTcF, QT, interval corrected for heart rate using Fridericia’s formula.

**FIGURE 3:**
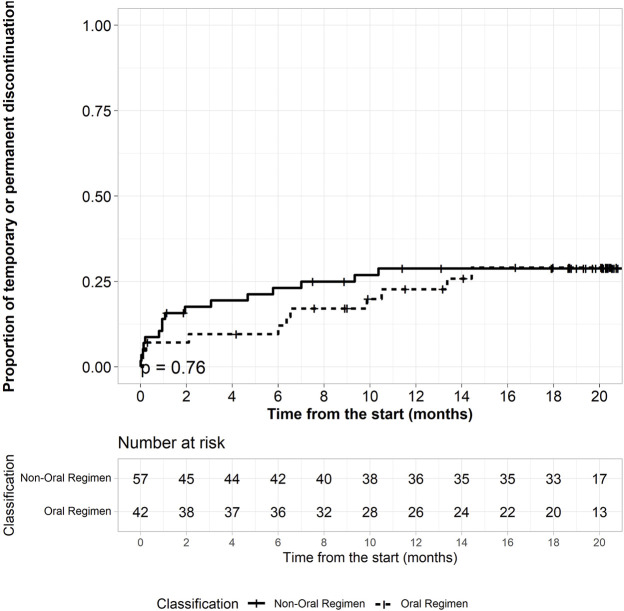
Time to temporary or permanent drug discontinuation by regimen.

No difference in terms of time to severe AE was observed between the two cohorts. Figure 3 shows the time to drug interruption (either temporarily or permanently) of both regimens, in the first 6 months of treatment: 6 (14.3%) patients in the BDQ-long group had to stop drugs due to AE compared to 15 (26.3%) on BDQ/SLI-long (*p* = 0.76). Median time to drug interruption in BDQ-long group was 6.4 months (IQR: 0.2–10.5) while it was 1 month (IQR: 0.2–5.5) in the other group (*p* = 0.09).

## Discussion

This cohort study of BDQ-based long regimens did not only assess the overall safety and effectiveness of BDQ-based long regimens among pre-XDR-TB patients but also determined the effect of regimen choice (BDQ-long or BDQ/SLI-long) on the QT prolongation effect, timing of having a severe AEs, drug discontinuation and time to conversion. To our best knowledge, this was the first comparison which has been made between all-oral and injectable-containing BDQ-based regimens in terms of both effectiveness and safety.

Regarding effectiveness assessment, compared to the globally reported 47% treatment success among patients treated for pre-XDR-TB (World Health Organization, 2020), BDQ-based long treatment regimens in this study showed high effectiveness among patients with highly resistant TB. Treatment success was similar for both BDQ-long and BDQ/SLI-long regimens. Overall, 76% success rate for BDQ-based long regimens among highly resistant TB was comparable with findings different settings (Barvaliya et al., 2020; Hatami et al., 2022; Ndjeka et al., 2018). In comparison with the reported treatment success rate of 47% among pre-XDR-TB patients in the program in Vietnam at the same period (Annual NTP report, 2019), the outcomes for BDQ-based long regimens reported in this study were superior. BDQ-based treatment should be used in all patients with highly resistant TB. The proportion with a favorable outcome was not significantly different between the BDQ-long and BDQ/SLI-long groups. Our sample size was not big enough to detect relatively small differences.

The reason that WHO recommends to restrict the use of SLI drugs in drug resistant TB was the concern about its high risk of ototoxicity and nephrotoxicity (Buziashvili et al., 2019; Shibeshi et al., 2019). Nevertherless, with an active drug safety monitoring system (aDSM), the early detection and management of AEs could mitigate eventual grave long-term consequences (World Health Organization, 2012; WHO, 2015). Taking into account the modest number of effective drugs left for pre-XDR-TB patients, SLI may still have a role to play, as long as its toxicity were adequately monitored. SLI can protect effective core drugs (such as BDQ, FQ) during the initial treatment period (van Deun et al., 2018), and prevent acquired resistance. Our sample was too small to assess this rare but important treatment outcome.

Both all-oral and SLI-containing BDQ-based long regimens had a similar high frequency of AEs as the proportion of patients having at least one severe AEs (grade 3–4) were 85.7% in BDQ-long and 84.2% in BDQ/SLI-long regimen. There was no significant difference between the two groups in terms of severe and serious QT prolongation. However, this AE tended to be more frequent in the BDQ-long group (52.4% with severe QT prolongation) in comparison with 38.6% in the BDQ/SLI-long group. This might be due to the difference in number of drugs with QT prolonging effect, as a significantly higher proportion of patients on BDQ-long had 3 QT prolonging drugs in the regimen, as the result of not using SLI in all-oral regimen. The combination of 3 anti-TB drugs with risk of QT prolongation (LFX_CFZ_BDQ) (Woosley et al., 2022) appeared in 26.2% (*n* = 11) of patients in the BDQ-long group, but only in 7.0% (*n* = 4) of patients in the BDQ/SLI-long group (*p* = 0.009). When constructing pre-XDR-TB regimens, it is essential to consider the effectiveness and safety of not only a single drug but also the combination of drugs. Other drugs than SLI can also cause severe AE (Borisov et al., 2019; Khan et al., 2022). Of WHO’s group A drugs, LZD was discontinued in 14.5% of patients having this drug in their regimen more often than other group A drugs (BDQ, LFX). The use of LZD increased significantly, as a substitute for SLI, and also as component of recently recommended RR-TB and pre-XDR-TB treatment regimens (BPaLM and BPaL) (World Health Organization, 2022). Subsequently, AEs and SAEs known to be caused by this drug, more specifically hematological and neurological AE, will also increase (Borisov et al., 2019; Sotgiu et al., 2012). Hence, even though all-oral BDQ-based long regimens can prevent toxicity due to injectable agents, active AE monitoring is still essential for these regimens.

There was no significant difference between BDQ-long and BDQ/SLI-long regimens groups in terms of time to severe AEs/SAEs and time to drug discontinuation. However, the BDQ/SLI-long regimen tended to have earlier drug interruption than BDQ-long regimen group. Median time to drug interruption in BDQ/SLI-long group was 1 month (IQR: 0.2–5.5) while it was 6.4 months (IQR: 0.2–10.5) in the other group (*p* = 0.09). Active drug safety monitoring using the CEM protocol resulted in an early detectionand informed the management of adverse effects that occured during treatment. Our previous study revealed that the risk of SLI related nephrotoxicity was associated with SLI dosing (Bao Ngoc et al., 2021). Therefore, the optimization of the dose of SLI should be considered to minimize the risk of toxicity. For LZD, neurological toxicity (peripheral and optic neuropathy) was usually seen after several months of use, median 5 months in the study by (Narita et al., 2007), thus, this drug could be considered for a limited time only, during the early treatment phase when high bactericidal activity is mostly required (van Deun et al., 2018).

We acknowledge that this study has its own strengths and limitations. One of the strengths was the use of the CEM protocol to prospectively record and report the AEs of patients during their entire treatment course, s (World Health Organization, 2012). Therefore, the safety information of all treatment regimens was well-captured, even for mild to moderate AEs, which were often under-reported by other studied reporting AEs (Borisov et al., 2019). The prospective design also led to comprehensive monitoring and rigorous data collection and cleaning. The limitation of our study was the relatively small sample size. Differences between the two regimens may have remained undetected. Moreover, multivariate analysis was not appropriate, and confounders could not be controlled for.

## Conclusion

Treatment success was similar for both all-oral and SLI-containing BDQ-based long regimens in patients with highly resistant TB. Both regimens had a similar high frequency of AEs. For both BDQ-long and BDQ/SLI-long regimens, active AEs monitoring is essential.

## Data Availability

The raw data supporting the conclusion of this article will be made available by the authors, without undue reservation.
