# Supplementary material for: Safety and effectiveness of all-oral and injectable-containing, bedaquiline-based long treatment regimen for pre-XDR tuberculosis in Vietnam
Source: Front Pharmacol. 2022 Oct 14;13:1023704. doi: 10.3389/fphar.2022.1023704 (PMC9614239; doi:10.3389/fphar.2022.1023704)
Supplement: Supplementary file 1 [file Table1.DOCX]

# Supplement Table S1. Schedule of follow-up examination

| **Month** | **Clinical examination** | **Chest Xray** | **Smear** | **Culture** | **DST 1 & 2** | **ECG** | | | **Liver function tests** | **Ure**  **Creatinine** | **Ion K, Mg, Ca** | **Lipase**  **Amylase** | **Bilirubin** | **HIV** | **Pregnancy** | **TSH** | **Visual test** | **Hearing test** |
| --- | --- | --- | --- | --- | --- | --- | --- | --- | --- | --- | --- | --- | --- | --- | --- | --- | --- | --- |
|  |  |  |  |  |  | **Reg. included others QT prolonged drugs** | **Reg. NOT included others QT prolonged drugs** | |  |  |  |  |  |  |  |  |  |  |
| **0** | **X** | **X** | **X** | **X** | **X** | **X** | | | **X** | **X** | **X** | **X** | **X** | **X** | **X** | **X** | **X** | **X** |
| **1** | **weekly** | **X** | **X** | **X** |  | **weekly** | | | **X** | **X** | **X** | **X** | **X** |  |  |  |  |  |
| **2** | **X** | **X** | **X** | **X** |  | **X** | | | **X** | **X** | **X** |  |  |  |  |  |  |  |
| **3** | **X** | **X** | **X** | **X** |  | **X** | | | **X** | **X** | **X** |  |  |  |  |  |  |  |
| **4** | **X** | **X** | **X** | **X** |  | **X** | | | **X** | **X** | **X** |  |  |  |  |  |  |  |
| **5** | **X** | **X** | **X** | **X** |  | **X** | | | **X** | **X** | **X** |  |  |  |  |  |  |  |
| **6** | **X** | **X** | **X** | **X** |  | **X** | | | **X** | **X** | **X** | **X** | **X** |  |  | **X** |  |  |
| **7** | **X** |  | **X** | **X** |  | **X** | |  |  | **X** |  |  |  |  |  |  |  |  |
| **8** | **X** |  | **X** | **X** |  | **X** | |  |  | **X** |  |  |  |  |  |  |  |  |
| **9** | **X** |  | **X** |  |  | **X** | | **X** | **X** |  | **X** |  |  |  |  |  |  |  |
| **10** | **X** |  | **X** |  |  | **X** | |  |  |  |  |  |  |  |  |  |  |  |
| **11** | **X** |  | **X** | **X** |  | **X** | |  |  |  |  |  |  |  |  |  |  |  |
| **12** | **X** | **X** | **X** |  |  | **X** | | **X** | **X** | **X** | **X** | **X** | **X** |  |  | **X** |  |  |
| **13** | **X** |  | **X** |  |  | **X** | |  |  |  |  |  |  |  |  |  |  |  |
| **14** | **X** |  | **X** | **X** |  | **X** | |  |  |  |  |  |  |  |  |  |  |  |
| **15** | **X** |  | **X** |  |  | **X** | | **X** | **X** | **X** | **X** |  |  |  |  |  |  |  |
| **16** | **X** |  | **X** |  |  | **X** | |  |  |  |  |  |  |  |  |  |  |  |
| **17** | **X** |  | **X** | **X** |  | **X** | |  |  |  |  |  |  |  |  |  |  |  |
| **18** | **X** | **X** | **X** |  |  | **X** | | **X** | **X** | **X** | **X** | **X** | **X** |  |  | **X** |  |  |
| **19** | **X** |  | **X** |  |  | **X** | |  |  |  |  |  |  |  |  |  |  |  |
| **20** | **X** |  | **X** | **X** |  | **X** | |  | **X** |  |  |  |  |  |  |  |  |  |
