# Supplementary material for: Safety and effectiveness of all-oral and injectable-containing, bedaquiline-based long treatment regimen for pre-XDR tuberculosis in Vietnam
Source: Front Pharmacol. 2022 Oct 14;13:1023704. doi: 10.3389/fphar.2022.1023704 (PMC9614239; doi:10.3389/fphar.2022.1023704)
Supplement: Supplementary file 2 [file Table2.DOCX]

**Supplementary Table S2. Number of patients with serious adverse events by regimen**

| **SAE^$^** | **BDQ/long**  **(N=42)** | | **BDQ/SLI-long**  **(N=57)** | | **Total**  **(N=99)** | |
| --- | --- | --- | --- | --- | --- | --- |
|  | **n** | **(%)** | **n** | **(%)** | **n** | **(%)** |
| **Frequency of SAE** |  |  |  |  |  |  |
| At least one SAE | 16 | (38.1) | 28 | (49.1) | 44 | (44.4) |
| At least two SAE | 5 | (11.9) | 13 | (22.8) | 18 | (18.2) |
| At least three SAE | 3 | (7.1) | 5 | (8.8) | 8 | (8.1) |
| **SAE by System Organ Class** |  |  |  |  |  |  |
| **Heart rate disorders** (prolonged QTcF) | 10 | (23.8) | 8 | (14) | 18 | (18.2) |
| **Any metabolic disorders** | 2 | (4.8) | 9 | (15.6) | 11 | (11.1) |
| Hyperuricemia | 1 | (2.4) | 3 | (5.3) | 4 | (4.0) |
| Hyperglycosemia | 1 | (2.4) | 6 | (10.5) | 7 | (7.1) |
| Increased amylase | 0 | (0.0) | 2 | (3.5) | 2 | (2.0) |
| **Any liver and biliary disorders** | 3 | (7.1) | 6 | (10.5) | 9 | (9.1) |
| Increased AST | 0 | (0.0) | 1 | (1.8) | 1 | (1.0) |
| Hepatitis | 3 | (7.1) | 4 | (7.0) | 7 | (7.1) |
| Increased direct bilirubin | 0 | (0.0) | 1 | (1.8) | 1 | (1.0) |
| Increased ALT | 0 | (0.0) | 1 | (1.8) | 1 | (1.0) |
| **Any electrolyte disturbances** | 3 | (7.1) | 7 | (12.3) | 10 | (10.1) |
| Hypocalcemia | 1 | (2.4) | 5 | (8.8) | 6 | (6.1) |
| Hypokalemia | 0 | (0.0) | 4 | (7.0) | 4 | (4.0) |
| Hypomagnesemia | 1 | (2.4) | 1 | (1.8) | 2 | (2.0) |
| Hyperkalemia | 1 | (2.4) | 0 | (0.0) | 1 | (1.0) |
| **Vision disorders** | 0 | (0.0) | 2 | (3.5) | 2 | (2.0) |
| **Gastrointestinal disorders** | 1 | (2.4) | 2 | (3.5) | 3 | (3.0) |
| **Psychiatric disorders** | 0 | (0.0) | 1 | (1.8) | 1 | (1.0) |
| **Hearing disorders** | 0 | (0.0) | 3 | (5.3) | 3 | (3.0) |
| **Renal disorders (Increased creatinine)** | 1 | (2.4) | 8 | (14) | 9 | (9.1) |
| **Hematologic disorders** | 4 | (9.5) | 0 | (0.0) | 4 | (4.0) |

$ definition of SAE: any death, hospitalization, life-threatening AE, permanent disability, or grade 4 AE.

# More than one SAE per patient possible

SAE, serious adverse event; AST, aspartate aminotransferase; ALT, alanine aminotransferase; QTcF, QT interval corrected for heart rate using Fridericia's formula.
